# Effect of compost and inorganic fertilizer on organic carbon and activities of carbon cycle enzymes in aggregates of an intensively cultivated Vertisol

**DOI:** 10.1371/journal.pone.0229644

**Published:** 2020-03-12

**Authors:** Zhanhui Zhao, Congzhi Zhang, Fang Li, Songfeng Gao, Jiabao Zhang

**Affiliations:** 1 State Experimental Station of Agro-Ecosystem in Fengqiu, State Key Laboratory of Soil and Sustainable Agriculture, Institute of Soil Science, Chinese Academy of Sciences, Nanjing, People’s Republic of China; 2 School of Surveying and Urban Spatial Information, Henan University of Urban Construction, Pingdingshan, People’s Republic of China; 3 College of Resources & Environment, Henan Agricultural University, Zhengzhou, People’s Republic of China; RMIT University, AUSTRALIA

## Abstract

**Background and aims:**

This paper was primarily devoted to understand the interactions of soil aggregates, organic carbon (C) and carbon cycle enzymes in aggregates under different fertilization managements, aiming to identify the effects of organic and inorganic fertilizer amendments on soil organic C accumulation and the activities of carbon cycle enzymes within aggregates in Vertisol.

**Methods:**

A Vertisol soil following 4-year compost and inorganic fertilizer amendments, i.e. no fertilizer (CK), mineral fertilizer (FR) and 60% compost N plus 40% fertilizer N (FRM), was collected to identify the dynamics of organic C, enzymes activities and their associations with macroaggregation using aggregate fractionation techniques.

**Results:**

The organic C content in all FR and FRM treatments was 8.24–41.15% higher than that in CK. An increased amounts of carbon cycle enzymes in aggregates or 0–20 cm bulk soil were also observed in FRM plots. Compared to FR, FRM significantly strengthened the structural stability of macroaggregates and the intimate connection between enzyme activities and macroaggregates.

**Conclusions:**

As a recommended measure, supplementation with organic manure such as compost strengthened the process of mutual promotion between carbon cycle enzymes and macroaggregates, and the synergistic effect would be highly beneficial to soil organic C sequestration.

## Introduction

Globally, soil organic C storage has been widely considered as a measure for mitigating global climate change through C sequestration in soils [1]. Soil organic C plays an important role as a pool of terrestrial C, in ecosystem productivity, in the functioning of agroecosystems and in cropland fertility [2,3]. Maintaining a satisfactory soil organic C content is particularly important for soil quality and sustaining the productivity of agro-ecosystems because it plays a decisive role in the cycle and transformation of nutrients by affecting soil physical, chemical, and biological properties [2,4,5]. Previous studies have shown that the accumulation rate of organic C in soils is strongly linked with the location of organic C within the soil matrix [6]. The accumulation pattern of organic C mainly depends on the characteristics of aggregates and the different types of organic C that accumulate in different aggregates [7]. New C accumulated more in macroaggregates (>250 μm) than in microaggregates (53–250 μm) and silt- and clay-sized aggregates (<53 μm), but decomposed faster in macroaggregates than in microaggregates [8]. Therefore, to understand the variation and prediction of the dynamics of carbon stocks in intensively cultivated Vertisols, a thorough understanding of the mechanisms by which organic carbon fractions are stabilized in soil is necessary.

Organic C accumulation was closely related to aggregation. Using the aggregate fractionation technique, soils are separated into the silt + clay fraction, microaggregates, small macroaggregates and large macroaggregates. And soil organic C can be distinguished from intermediate and passive organic C pools into different active C pools through fractionation [9]. According to the hierarchical aggregate model of Tisdall and Oades [10], the silt + clay fraction is mainly an association of primary particles with bacteria and mucilages through H-bonding. Microaggregates are first formed freely and then serve as building blocks for the formation of macroaggregates under the cementing effect of microbial exudates, fungal hyphae, and particulate organic matter. Similarly, macroaggregate-sized particles combine to form larger macroaggregate structures. In the process, macroaggregate-sized particles consist of more labile particulate organic matter (POM) C, whereas microaggregates and the silt + clay fraction contain more recalcitrant organic C that is physically protected from decomposition by mineral particles that accumulate on and around the surface of organic C [10,11]. Aggregation results from the rearrangement, flocculation and cementation of particles. Organic C acts as a binding agent and as a nucleus in the formation of aggregates [12,13]. Thus, aggregate turnover and organic C accumulation are inextricably tied and act at the aggregate size scale.

The variation of soil organic C concentration was affected by soil microbial activities as well as fertilization and other tillage activities. The input from vegetation residues, anthropogenic inputs such as compost, manure, and soil management, such as agricultural tillage, have a decisive influence on the accumulation and chemical composition of soil organic C [14,15]. Whereas these inputs tend to accelerate carbon sequestration, tillage and aeration favour organic C decomposition and thus cause C loss. The stability of organic C in soils is generally dependent on resistance to microbial decomposition [16]. It is well recognized and documented that the activities of enzymes produced by microorganisms are potentially sensitive indicators of change in the biochemical composition of the soil organic C content [17]. The soil enzymes involved in C cycling mainly include cellulase, invertase, urease and catalase [18,19]. Extracellular enzymes such as cellulase are produced by fungi during the decomposition of vegetation residues and other cellulase compounds [20]. In intensively cultivated cropland, the returned vegetation residues contain numerous cellulase polymer substrates, which are the major accumulated soil organic C [21]. Cellulase hydrolysis into glucose is mainly achieved by the complex enzyme cellulase [22]. Some polysaccharide macromolecular compounds, such as sucrose, are further hydrolysed under the catalytic action of invertase [19]. Soil urease catalyses the hydrolysis of phthalein bonds in organic molecules and promotes the transformation of soil organic nitrogen into available nitrogen, which is an important nitrogen source for plants and microorganisms [23]. In addition, through enzymatic hydrolysis reactions, catalase can remove the toxic effect of hydrogen peroxide, which is produced by biological respiration and biochemical oxidation of organic matter, on soil and organisms [24]. Catalysed by soil enzymes and through a series of complex chemical reactions, macromolecular organic compounds are ultimately degraded into available C and N to promote nutrient cycling.

Previous studies have widely demonstrated that compost and inorganic fertilizer amendment alter the distribution of soil aggregates and stabilize organic C [7,25,26]. The application of inorganic fertilizers was reported to affect the soil aggregate distribution and its associated organic C by increasing the aboveground and root biomass due to the immediate supply of plant nutrients in sufficient quantities [27]. Organic manures act as a humic and semi-humic C source, directly providing organic C in the soil, which helps to sequester C [28]. However, under compost and inorganic fertilizer application, knowledge about the coordination mechanism between aggregate and organic C is still limited. In particular, little is known about how organic C cycling occurs in soil aggregates, which strongly limits our understanding of micro-scale soil structure dynamics. Notably, soil enzymes respond to soil management practices and act as good indicators of organic matter decomposition and nutrient cycling [19]. Therefore, by studying the relationship between organic C and the activities of carbon cycle enzymes in aggregates, soil aggregate formation and C sequestration mechanisms may be revealed.

To date, most studies have focused on the relationship between soil enzyme activity and soil fertility, or the response of enzyme activity to fertilization, but there are only a few reports on enzyme activities within aggregates under compost and inorganic fertilizer application in China. Our previous study showed that compost application significantly increased macroaggregate (>250 μm) formation and that macroaggregates played a key role in organic C accumulation in our tested soils. However, the contributions of aggregate-associated organic C to the total soil organic C concentration at the 0- to 20-cm depths were different [29]. Therefore, to understand the interactions of soil aggregates, organic C and C cycle enzymes, an attempt has been made to determine the effects of compost and inorganic fertilizer on (1) the processes of organic C accumulation at the aggregate scale by measuring changes in subfraction-associated organic C and (2) the biochemical characteristics of accumulated organic matter at the aggregate scale by measuring the activities of C cycle enzymes in soil and separated aggregates under compost and inorganic fertilizer treatments. We hypothesized that compost and inorganic fertilizer amendments would increase soil organic C by improving macroaggregation and strengthen the interaction between aggregates and enzyme activities.

## Material and methods

### Experimental site

With the support of Institute of Soil Science, Chinese Academy of Sciences, the study was conducted in a fertilized experimental field (latitude 33°33´ N, longitude 114°02´ E) situated in Xiping County, Henan Province, China. The experiment started on June 2012 in a well-drained field, a typical irrigable cropland in the North China plain. The average annual temperature and precipitation in this area are 14.8 °C and 852 mm, respectively. The average sunshine duration and the average frost-free period are 2659 hours and 121 days, respectively. In June 2012, the total organic C and N were 5.92 g/kg soil and 0.57 g/kg soil, respectively. The soil, derived from a fluviolacustrine deposit plain, has a loam texture, with 0.10 kg sand/kg soil, 0.9.15 kg silt/kg soil, and 0.45 kg clay/kg soil. Immediately after wheat (Triticum aestivum L.) or maize (Zea mays L.) harvest, all the surface crop straw was crushed by mechanical equipment and then return to the field for all treatments. Fertilizers were broadcasted evenly onto the soil surface before plowing (mechanized farming to a depth of 20 cm soil) for all fertilization treatments, and the same tillage was also applied in those plots without fertilization.

### Experimental design

The field experiment was established in June 2012 in a well-drained field where wheat (*Triticum aestivum* L.) was grown in winter and maize (*Zea mays* L.) was grown in summer. The site is in a region typical of the North China Plain. A randomized block design was used to prepare three replicates of each of four treatments: pre-soil, no fertilizer, i.e., control (CK), NPK fertilizer (FR), and 60% compost plus 40% nitrogen (N) fertilizer (FRM). Calcium superphosphate (39 kg P/ha in the treatment with NPK) and potassium sulfate (75 kg K/ha in the treatment with NPK) were applied to each crop as basal fertilizers; compost (135 kg N/ha for FM) was applied annually as a basal fertilizer to maize. Urea totalled 225 and 240 kg N/ha in the maize and wheat NPK treatments, respectively. Organic manure, phosphorus (P), and potassium (K) fertilizers were applied to the soil before sowing, and nitrogenous fertilizer was applied at the rates of 40% and 60% of the total amount before sowing and during the elongation stage, respectively. In the FM treatment, the compost addition rate was 6.32 t/ha, which corresponds to approximately 1.50 t OC/ha. Urea was applied as a supplemental fertilizer at a rate of 90 and 240 kg N/ha for maize and wheat, respectively. Calcium superphosphate and potassium sulfate were used to supplement insufficient levels of phosphorus and potassium. No fertilizer or compost was applied in the CK treatment.

### Soil sampling and aggregate fractionation

In June 2016, immediately after the wheat harvest, four soil samples were randomly collected in the 0–20 cm soil layer at different locations in each plot using a stainless-steel soil sampler. Pre-soil samples were also collected for testing in June 2012. Soil samples were wet-sieved into large macroaggregates (> 2000 μm), small macroaggregates (250–2000 μm), microaggregates (53–250 μm), and the silt + clay fraction (< 53 μm) at ambient temperature according to the method described by Elliott [30]. After separation, the aggregate fractions were dried at 40 °C for soil property analysis or, using the density fractionation method, for further fractionation of macro- and microaggregates into different subfractions, including coarse intraparticulate particulate organic matter (Coarse iPOM; > 250 μm), fine intraparticulate particulate organic matter (iPOM, 53–250 μm), and the silt + clay subfraction (SC, < 53 μm). Fifty grams of macro- or microaggregates were placed into 250 ml centrifuge tubes, and 150 ml of 1.85 g cm^-3^ ZnBr solution was added. After centrifugation and filtration, free particulate organic matter (fPOM) was obtained. fPOM was further resuspended in 150 ml of sodium hexametaphosphate (0.5%, w/v) solution, and shaken for 18 h to completely disperse aggregates. Finally, the samples were sieved through 250- and 53-μm sieves to obtain coarse iPOM, fine iPOM, and silt + clay subfractions. Yu et al. [31] reported the fractionation method and results in detail.

### Determination of organic carbon and enzyme activities

After separation, the aggregate fractions and another soil subsample of the whole soil were used to determine total organic C and soil enzymes. The organic C content in the soil and aggregates was determined by the wet oxidation-redox titration method [32]. Cellulases were determined according to Hope and Burns [33]. Cellulases are enzyme systems that degrade cellulose and release reducing sugars as the end product, and the reducing sugars were determined by the anthrone colorimetric analysis method. In the context of this work, the term refers to the combined action of endoglucanase (EC 3.2.1.4), exoglucanase (EC 3.2.1.91) and ᵝ-D-glucosidase (EC 3.2.1.21) on Avicel, a purified depolymerized alpha cellulase. Invertase (EC 3.2.1.26) activity was determined as suggested by Schinner and von Mersi [34]. One gram (on an oven-dried basis) of soil or aggregates was incubated with 1.2% (w ⁄v) sucrose solution and acetate buffer (pH 5.5) in the dark at 50 °C for 3 h. After incubation, the contents were filtered, and 1 ml of the filtrate was used to estimate the amount of reducing sugars using the 3,5-dinitrosalicylic acid (DNS) method [35]. The urease (EC 3.5.1.5) activity in the soil was measured with the buffered method of Kandeler and Gerber [36]. Briefly, 0.5 mL of a solution of urea (0.48%) and 4 mL of borate buffer (pH 10) were added to 1 g of soil in hermetically sealed flasks and then incubated for 2 h at 37 °C. The ammonium content of the centrifuged extracts was determined by spectrophotometry at 578 nm by a modified indophenol blue reaction. Catalase (EC 1.11.1.6) activity was measured by back-titrating residual H_2_O_2_ with KMnO_4_ [37,38]. Two grams of soil samples were added to 40 mL distilled water with 5 mL of 0.3% hydrogen peroxide solution. The mixture was shaken for 20 min, and then 5 mL of 1.5 mol/L H_2_SO_4_ was added. Afterwards, the solution was filtered and titrated using 0.02 mol/L KMnO_4_. The reacted amount of 0.02 mol/L KMnO_4_, calculated per gram of dry soil, was used to express the activity of catalase. Enzyme activities were measured in soil samples of each treatment, with controls made by mixing buffer with either soil fractions or substrate solution. The values were corrected by subtracting the combined absorption values for the sample and substrate controls from those for the analytical samples. The activity of cellulase and invertase was expressed in mg sugars released/g dry soil (37°C 24 h). The activity of urease and catalase was expressed as μg NH_3_-N/g dry soil (37°C 24 h) and μmol H_2_O_2_/g dry soil (25°C 24 h).

### Statistical data evaluation

Statistical analysis was performed using SPSS v. 16.0 software (SPSS Inc., Chicago, USA). One-way analysis of variance (ANOVA) followed by a least significant difference (LSD) test was used to compare differences between the different treatments at significance levels of *P* < 0.05. Redundancy analyses (RDA) [39] were used to evaluate the relationships between soil aggregates, organic C content and soil enzyme activities in soil or aggregates using the R statistical package (MathSoft, Inc., Cambridge, Massachusetts).

## Results

### Soil organic carbon concentration and enzymes activities in bulk soil

**Fig 1:**
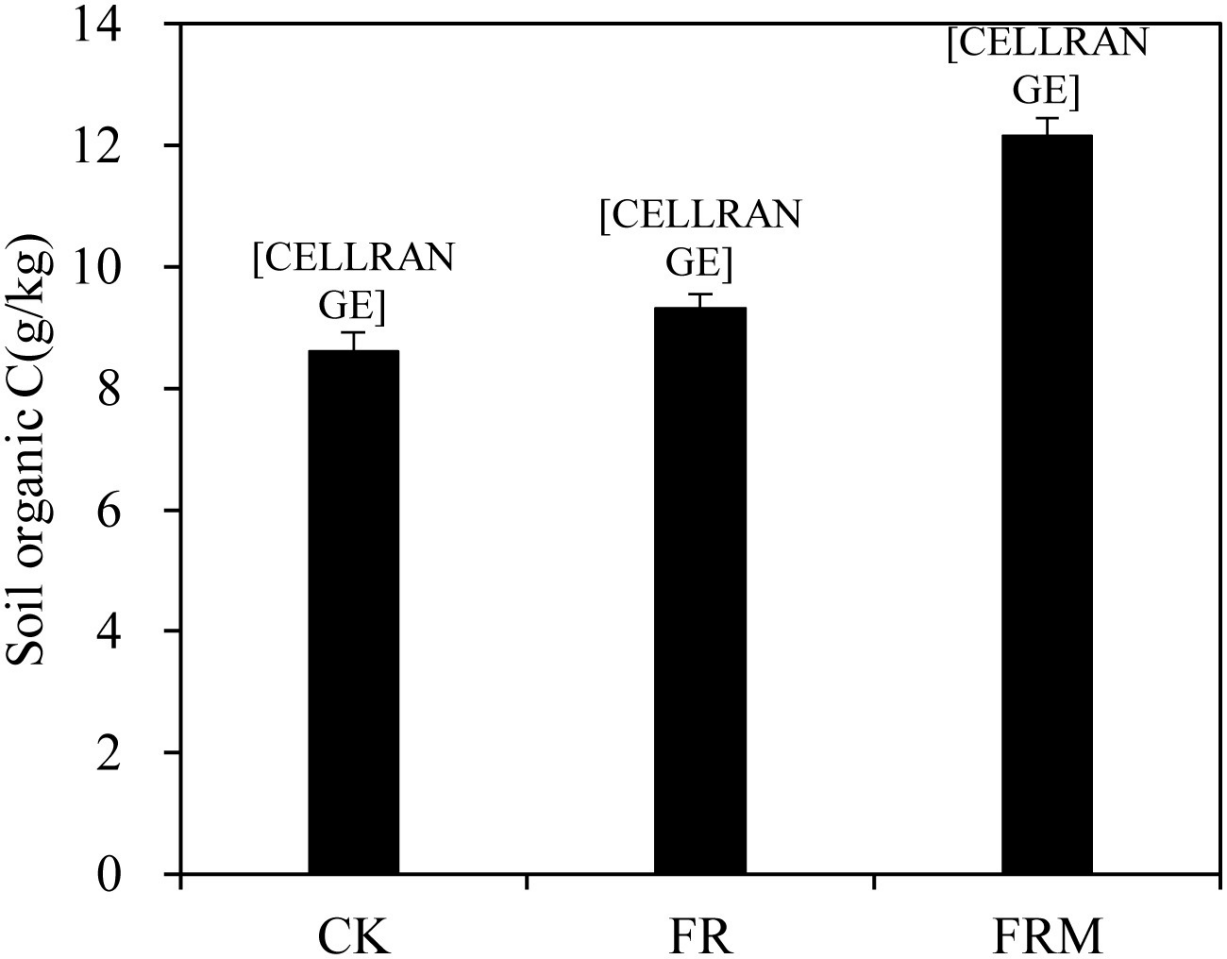
Compost and inorganic fertilizer effects on the organic C content in the soil. Vertical bars denote the standard error of the mean (n = 3). Different letters a, b and c indicate significant differences between treatments at *P* < 0.05.

During the four-year duration of the experiment, the soil organic C concentration in the control plot, which did not receive any type of fertilizer, was the lowest compared to the soil organic C concentration in the fertilization treatment. The soil organic C concentrations in the FR and FRM treatments were 9.32 and 12.15 g/kg, respectively, and increased by 8.24% and 41.15%, respectively, compared with CK (Fig 1). What’s more, fertilization also helped to increase crop biomass, which in turn increased the amount of straw returned to the field. Our previous results have proved that organic fertilizers could increase more crop production than inorganic fertilizers [40]. Thus, the real cause of organic C variation lied that a single application of chemical fertilizer significantly improved soil organic C accumulation, but the co-application of compost and inorganic fertilizer resulted in the greatest increase in organic C in the present research.

**Fig 2:**
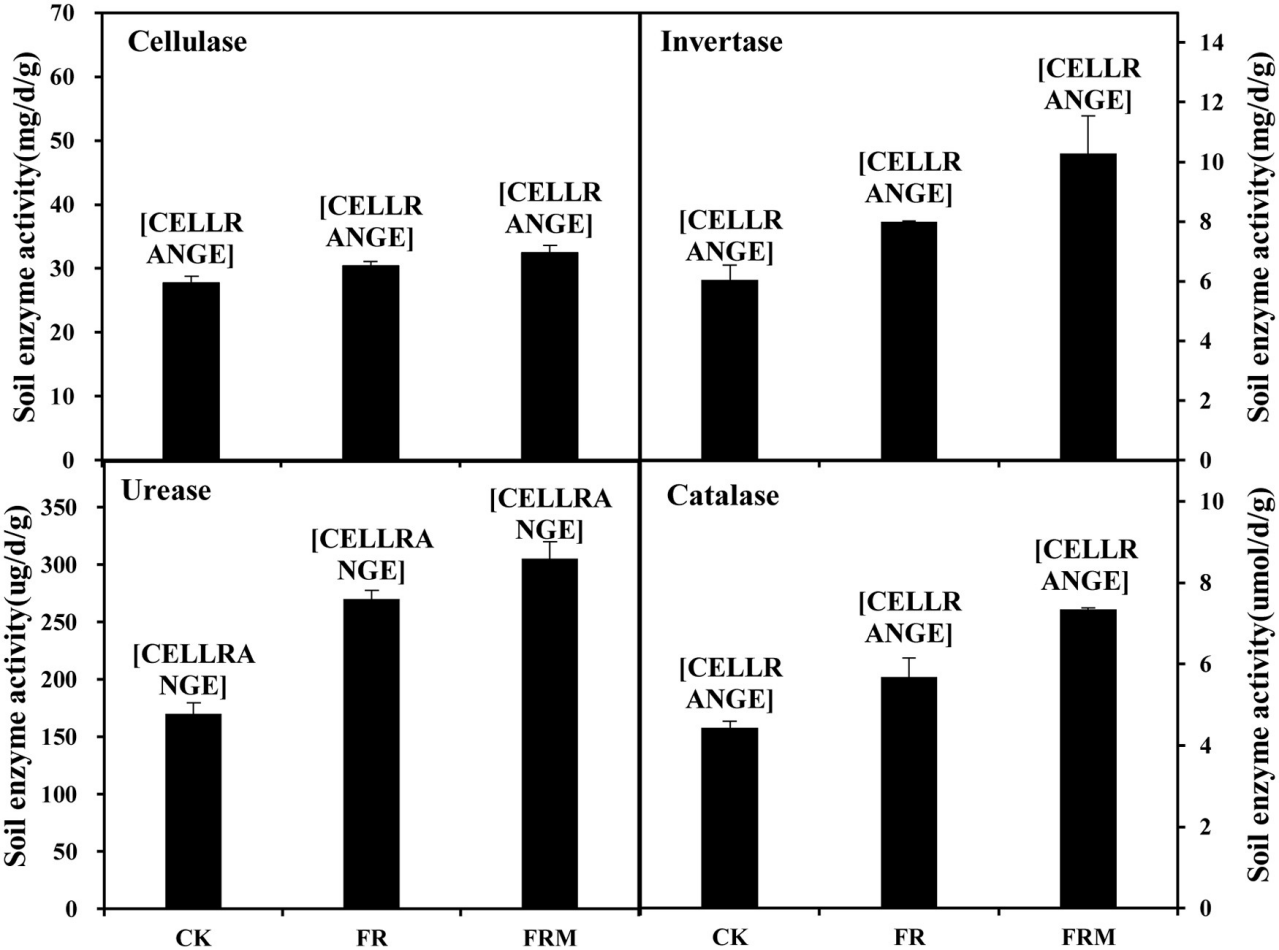
Compost and inorganic fertilizer effects on cellulase, invertase, urease and catalase activities in soil. Vertical bars denote the standard error of the mean (n = 3). Different letters a, b and c indicate significant differences between treatments at *P* < 0.05.

The cellulase, invertase, urease and catalase activities were significantly influenced by the different fertilization treatments (Fig 2). The activities of the four enzymes in the compost or inorganic fertilization treatment were significantly higher than those in the CK treatment. Compared with CK, FR application increased cellulase, invertase, urease and catalase activities by 9.50, 32.33, 58.99, and 28.04%, respectively. However, the activities of the four enzymes in the FRM treatment were higher than those in CK or FR. Thus, substituting compost for chemical nitrogen application stimulated soil enzyme activities markedly on the basis of the application of a single mineral fertilizer.

### Aggregate mass distribution and organic carbon concentration in aggregates

**Fig 3:**
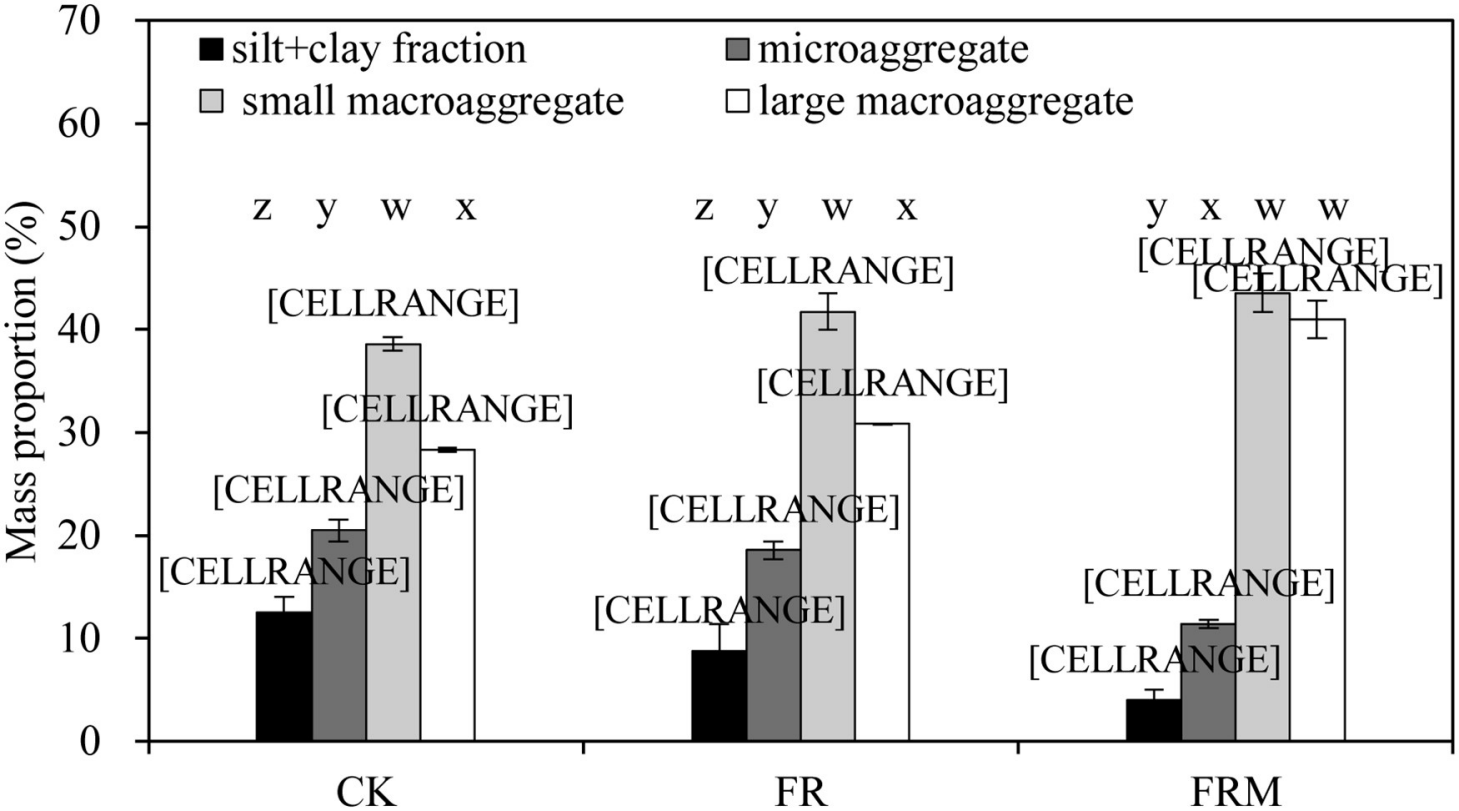
Mass proportion (%) of soil aggregates in 0- to 20-cm bulk soil affected by compost and inorganic fertilizer. Vertical bars denote the standard error of the mean (n = 3). Different letters a, b and c indicate significant differences between treatments for the same aggregate at *P* < 0.05. Different letters w, x, y and z denote significant differences between aggregates for the same treatment at *P* < 0.05.

The microaggregates accounted for 311.41–20.50% of the total soil mass in all treatments (Fig 3). The mass proportion of the free silt + clay fraction was 4.01–12.55%. Chemical fertilizer application alone (FR) or with the addition of compost (FRM) significantly (*P* < 0.05) reduced the mass proportion of microaggregates and the free silt + clay fraction. However, substituting compost for fertilizer (FRM) significantly (*P* < 0.05) increased the mass proportion of small macroaggregates and large macroaggregates by 12.90 and 44.66%, respectively. The mass proportion of large macroaggregates also presented a significant increase in FR. The analysis results also showed that the mass proportion of macroaggregates occupied a dominant position in the whole soil mass under the same treatment conditions. The present research indicated that after several years of compost or inorganic fertilizer treatments, the distribution characteristics of aggregates changed mainly through an increase in the number of macroaggregates (>250 μm) and a decrease in the number of microaggregates (<250 μm).

**Fig 4:**
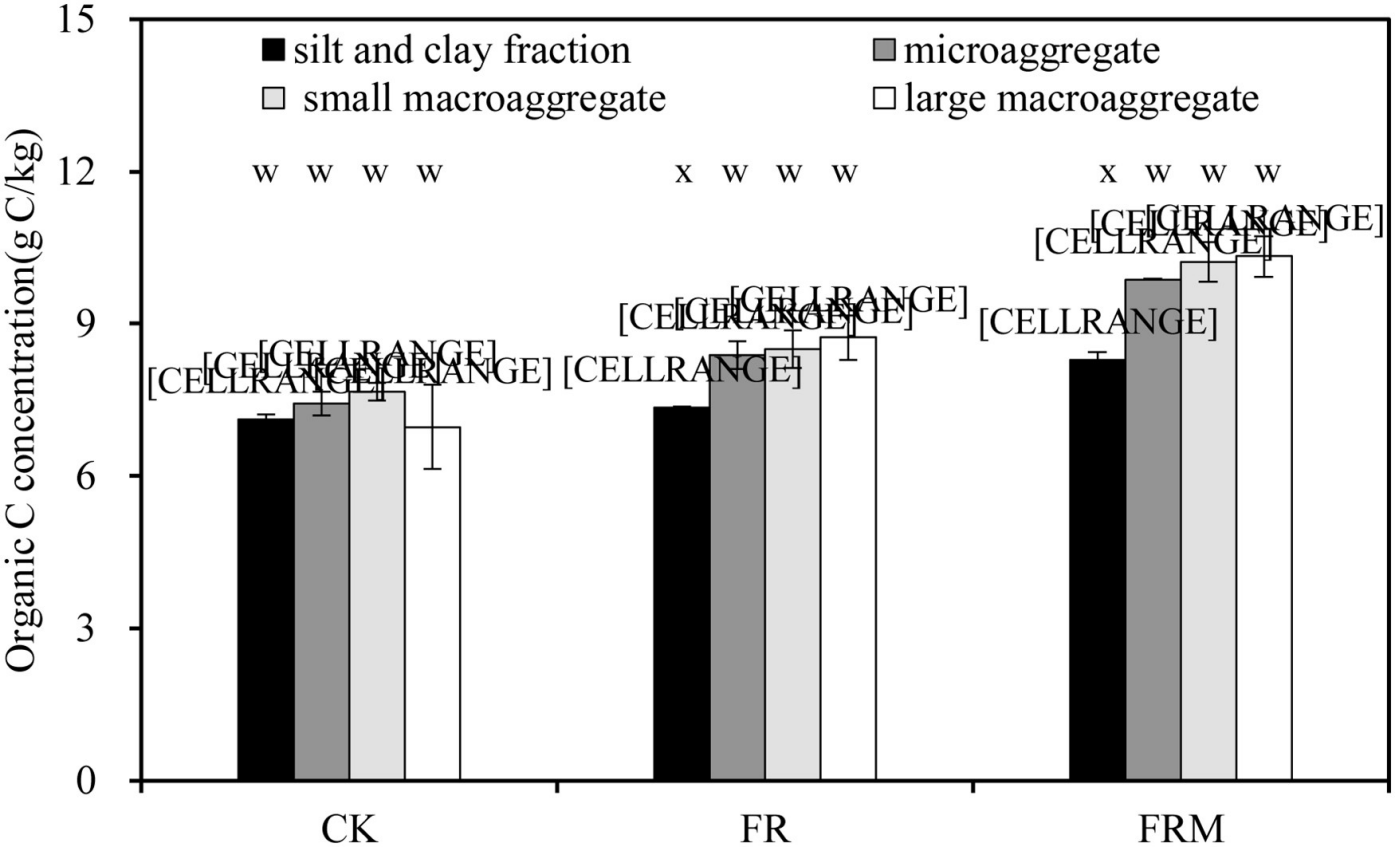
Soil aggregate-associated organic C in the 0- to 20-cm bulk soil affected by compost and inorganic fertilizer. Vertical bars denote the standard error of the mean (n = 3). Different letters a, b and c indicate significant differences between treatments for the same aggregate at *P* < 0.05. Different letters w and x denote significant differences between aggregates for the same treatment at *P* < 0.05.

Aggregate-associated organic C in FRM ranged from 8.29 to 10.33 g C/kg aggregate and was significantly (*P* < 0.05) higher than the value in FR, which ranged from 7.34 to 8.72 g C/kg. CK presented the lowest values for organic C in aggregates (Fig 4). The traditional single application of chemical fertilizer enhanced aggregate-associated organic C significantly, but the additional compost application (FRM) further increased the organic C in aggregates relative to the original value. The analysis results also revealed that macroaggregates (>250 μm) were associated with more organic C than microaggregates (<250 μm) under the same treatment.

When large and small macroaggregates were further separated into coarse/fine iPOM and the silt + clay subfraction (mSC or MSC), compared to CK, chemical fertilizer application alone (FR) solely improved fine iPOM in macroaggregates (>250 μm) at the significance level *P* < 0.05, whereas substituting compost for fertilizer (FRM) signally increased the organic C in the silt + clay subfraction and fine iPOM in both microaggregates (53–250 μm) and macroaggregates (>250 μm) (Table 1).

**Table 1:** Organic C (g C/kg aggregate) in subfractions within macroaggregates and microaggregates in the 0- to 20-cm soil layer affected by compost and inorganic fertilizer.

Treatments	Microaggregates (53–250 μm)		Macroaggregates (>250 μm)		
	silt + clay subfraction (mSC, <53 μm)	fine iPOM (53f, 53–250 μm)	silt + clay subfraction (MSC, <53 μm)	fine iPOM (250f, 53–250 μm)	Coarse iPOM (250c,>250 μm)
CK	7.31±0.36b	11.05±0.59c	8.76±0.57b	7.62±0.33c	11.64±0.3a
FR	7.86±0.37b	12.38±0.28b	9.39±0.3b	9.02±0.1b	11.8±0.19a
FRM	9.14±0.53a	13.71±0.28a	11.43±0.13a	12.37±0.22a	12.02±0.31a

Values are means (n = 3) with standard error. Different letters within the same column indicate significant differences between treatments at *P* < 0.05.

Organic C concentrations in the free silt + clay fraction and microaggregates were correlated with the mass proportion of large macroaggregates (*P*<0.001) (Eq 2) and with the mass proportion of large plus small macroaggregates (*P*<0.001) (Eq 3), However, there was no significant correlation with the mass proportion of small macroaggregates (*P* = 0.06) (Eq 1). Thus, organic C in microaggregates (<250 μm) might be crucially linked to the formation of macroaggregates.

$$\text{mass}\;\text{proportion}\;\text{of}\;\text{small}\;\text{macroaggregates}=-3.71{\text{SC}}_{\text{scf}}+3.56{\text{SC}}_{\text{m}}+38.89,{\text{R}}^{2}=0.61,P=0.06$$ (1)

$$\text{mass}\;\text{proportion}\;\text{of}\;\text{large}\;\text{macroaggregates}=11.22{\text{SC}}_{\text{scf}}-0.25{\text{SC}}_{\text{m}}-49.53,{\text{R}}^{2}=0.99,P<0.001$$ (2)

$$\text{mass}\;\text{proportion}\;\text{of}\;\text{large}\;\text{plus}\;\text{small}\;\text{macroaggregates}=7.5{\text{SC}}_{\text{scf}}+3.32{\text{SC}}_{\text{m}}-10.63,{\text{R}}^{2}=0.99,P<0.001$$ (3)

### Enzyme activities in aggregates

**Fig 5:**
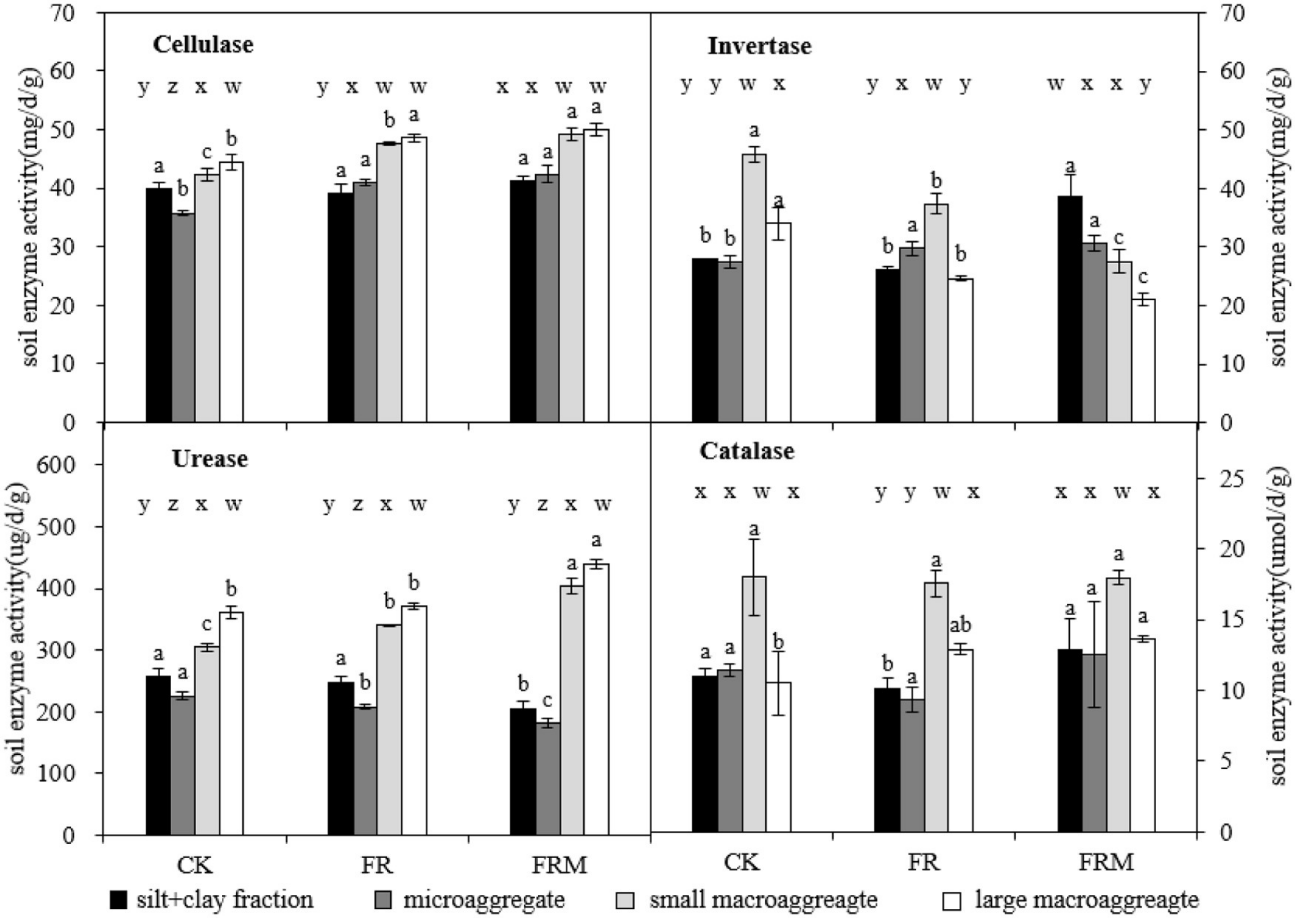
Compost and inorganic fertilizer effects on cellulase, invertase, urease and catalase activities in soil aggregates. Vertical bars denote the standard error of the mean (n = 3). Different letters a, b and c indicate significant differences between treatments at *P* < 0.05. Different letters w, x, y and z denote significant differences between aggregates for the same treatment at *P* < 0.05.

Cellulase or urease in large or small macroaggregates was higher active than that in silt and clay fractions and microaggregates. Substituting compost for fertilizer (FRM) significantly strengthened macroaggregate-associated cellulase and urease activities but weakened urease activities in the microaggregates and silt + clay fraction (Fig 5). The distribution characteristics of invertase activities in aggregates with different particle sizes were significantly changed by different fertilizer treatments; specifically, there was an increase in invertase activity in large and small macroaggregates and a reduction in that in the silt and clay fraction (<53 μm) and microaggregates (53–250 μm) in the FRM treatment. Compared to CK, chemical fertilizer application alone (FR) enhanced catalase activity only in the silt and clay fraction (<53 μm), while FRM significantly improved catalase activity only in large macroaggregates.

### Relationships between aggregates and organic C or enzyme activities in aggregates

Compared with microaggregates (<250 μm) and small macroaggregates (250–2000 μm), large macroaggregates were more strongly correlated with the organic C content in 0- to 20-cm bulk soil. Significant relationships were found between macroaggregates (>250 μm) and fine iPOM (53f or 250f) or the silt + clay subfraction (mSC or MSC), except for coarse iPOM (Table 2). Therefore, the silt + clay subfraction and fine iPOM might play a vital role in the formation of microaggregates (53–250 μm) and macroaggregates (>250 μm). Moreover, cellulase, invertase, urease and catalase activities were also strongly related to small or large macroaggregates; however, the relationship between carbon cycle enzymes and microaggregates (<250 μm) was not significant (Table 3). The result revealed that carbon cycle enzymes were closely related to the amount of macroaggregates (>250 μm).

**Table 2:** Relationships between organic C contents in physical subfractions (y) and mass proportion (%) of soil aggregates (x) in the 0- to 20-cm layer.

C fraction	Silt and clay fraction (<53 μm)		Microaggregates (53–250 μm)		Small macroaggregates (250–2000 μm)		Large macroaggregates (>2000 μm)	
	Equation	R^2^	Equation	R^2^	Equation	R^2^	Equation	R^2^
OC	y = 0.04x^2^-x+15.08	0.73	y = 0.02x^2^-1.15x+22	0.91*	y = 0.01x^2^-0.09x+0.87	0.69	y = -0.002x^2^+0.41x-2	0.99**
**mSC**	y = -1.16ln(x)+10.45	0.51	y = -2.73ln(x)+16	0.70	y = 0.32x-5.08	0.84*	y = 0.14x+3.32	0.88*
**53f**	y = -1.73ln(x)+15.87	0.62	y = -0.24x+16.47	0.72	y = -0.04x^2^+4.02x-80	0.89*	y = -0.02x^2^+1.61x-18	0.91**
**MSC**	y = 0.041x^2^-0.93x+14	0.73	y = 0.03x^2^-1.24x+22	0.87*	y = 0.41x-6.91	0.69	y = 0.21x+2.99	0.93**
**250f**	y = -3.47ln(x)+16.65	0.81*	y = -0.49x+17.84	0.93**	y = 28.40ln(x)-95.95	0.68	y = -0.02x^2^+1.72x-26	0.99**
**250c**	y = 0.01x^2^-0.11x+12	0.11	y = 0.01x^2^-0.26x+14	0.23	y = 0.1x+7.81	0.73	y = 0.03x+10.75	0.42

* and ** indicate regression analysis reached significance at *P* < 0.01 and *P* < 0.05, respectively.

**Table 3:** Relationships between cellulases, invertase, urease and catalase activities (y) and mass proportion (%) of soil aggregates (x) in the 0- to 20-cm layer.

Enzyme activity	Silt and clay fraction (<53 μm)		Microaggregates (53–250 μm)		Small macroaggregates (250–2000 μm)		Large macroaggregates (>2000 μm)	
	Equation	R^2^	Equation	R^2^	Equation	R^2^	Equation	R^2^
Cellulase	y = 0.01x^2^-0.62x+34.31	0.53	y = -0.01x^2^+0.03x+33.95	0.63	y = 34.77ln(x)-99.12	0.93**	y = -0.03x^2^+2.46x-17.38	0.84*
Invertase	y = -2.79ln(x)+13.73	0.61	y = -0.02x^2^+0.29x+9.77	0.75	y = 29.36ln(x)-101.09	0.85*	y = 11.03ln(x)-30.46	0.89*
Urease	y = -0.74x^2^+0.11x+312	0.67	y = -2.08x^2^+54.18x-42.67	0.71	y = -2.96x^2^+268.06x-5760	0.91**	y = -1.61x^2^+122.47x-1992	0.86*
Catalase	y = -1.97ln(x)+9.78	0.71	y = -4.26ln(x)+17.70	0.83*	y = -0.04x^2^+3.66x-79.27	0.82*	y = 7.27ln(x)-19.61	0.91**

* and ** indicate regression analysis reached significance at *P* < 0.01 and *P* < 0.05, respectively.

OC denotes the organic C content in 0- to 20-cm bulk soil. MSC, 250f and 250c denote the respective silt + clay subfraction, fine iPOM and coarse iPOM-associated C within macroaggregates. mSC and 53f denote the respective silt + clay subfraction and fine iPOM-associated C within microaggregates

## Discussion

### Effect of fertilization on organic C and stability of aggregate

Plant litter materials provide the primary substrates for organic matter formation in soil, and their composition and properties are essential controlling factors for the transformation of soil organic matter [41]. Fragments of plants and soil fauna are first broken up into small pieces at the onset of decomposition, and the initial decomposition products are further degraded into small biopolymers. Then, exogenous organic materials are transformed into humic substances, which are an important component of organic matter, under a suite of transformation processes [42]. Fertilization with chemical fertilizer is considered the most effective way to increase both aboveground yield and belowground root biomass within the shortest possible time. Zhang et al. [43] also reported that fertilization had positive effects on soil organic C sequestration since fertilization could indirectly increase the original C from crop residues above or below ground. However, compared with organic fertilizers, chemical fertilization might not an ideal practice from the point view of C sequestration in soil, mixed application of organic and inorganic fertilizers was a compromise between crop yields increase and soil C sequestration by balancing soil nutrients and adding soil organic matter directly from organic fertilizer [14]. Organic inputs, such as organic manure or compost, which mainly originate from a mixture of organic compounds produced by livestock manure or heap-rot of plant remains, result in the direct input of abundant humic carbon or humus into soil. Organic fertilizer is increasingly being used to increase soil fertility by improving chemical and biological soil properties. Thus, the application of organic manure might shorten the soil humus process and accelerate the accumulation of humus carbon sources. In the present research, similar to previous results, compared to CK, the FRM treatment enhanced the content of soil organic C by 41.15%, which was approximately five times higher than the increase resulting from chemical fertilizer application alone (FR) in the field experiment during the past five years.

**Fig 6:**
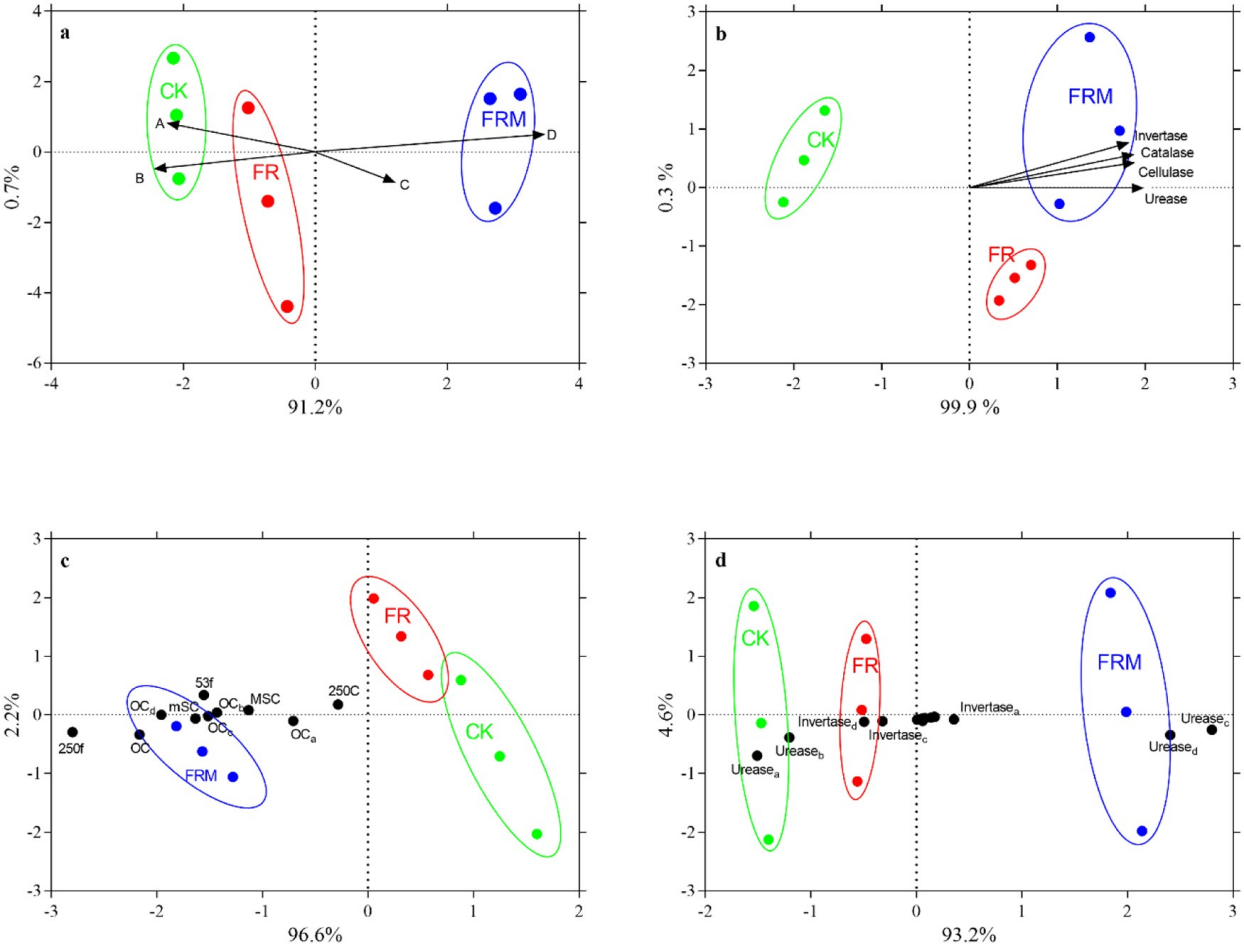
Redundancy analysis (RDA) relating organic C, mass proportion of aggregates or enzyme activities to different fertilization treatments in 0- to 20-cm bulk soil. CK, FR and FRM indicate no fertilizer, chemical fertilizer application alone and the substitution of compost for fertilizer, respectively. A, B, C and D indicate the mass proportion of the silt and clay fraction (<53 μm), microaggregates (53–250 μm), small macroaggregates (250–2000 μm) and large macroaggregates (>2000 μm), respectively. The lowercase letters a, b, c and d on the lower right of the term indicate organic C or enzymes within the silt and clay fraction (<53 μm), microaggregates (53–250 μm), small macroaggregates (250–2000 μm) and large macroaggregates (>2000 μm), respectively.

Aggregate stability is an important indication of soil structural development, C dynamics, and other soil processes. Organic fertilization strongly enhanced the formation of macroaggregates (>250 μm) and reduced the mass proportion of microaggregates (<250 μm) (Figs 3 and 6a), which suggests that organic fertilizer might have a very strong effect on the silt + clay fraction or microaggregates to assemble macroaggregates (>250 μm). The mechanism of organic C stabilization in many soils depends on the physical protection capability of aggregates according to some recent reports [7,8]. Some scholars have proposed that the free silt + clay fraction might bind together with free particulate organic matter to form microaggregates or bind directly with microaggregates to form macroaggregates, and the differences in aggregate turnover largely control the difference in fine iPOM [9,31]. Bhattacharyya et al. [44] demonstrated that the addition of mineral fertilizer only increased the organic C concentration in the free silt + clay fraction and not the mass proportion of aggregates. However, in the present study, mineral fertilizer application had no significant effect on the silt + clay subfraction, but the addition of manure fertilizer enhanced both the silt + clay subfraction (mSC, MSC) and fine iPOM (53f, 250f) (Table 1). Soil aggregates, as the main storage sites of soil organic C, can incorporate organic C through their own physical protection to prevent decomposition by microorganisms. The resulting macroaggregates could contain more organic C, while the silt + clay fraction and microaggregates might assemble a higher proportion of older C due to its indestructible structure. Based on this theory, humus input into the soil by organic fertilizer first combined with the silt + clay fraction or microaggregates to form macroaggregates, as shown in Fig 3. The application of manure fertilizer stimulated the silt + clay fraction, and microaggregates assembled more organic C to form larger aggregate particles and significantly improved aggregate stability (*P* < 0.05).

### Effect of fertilization on enzyme activities in aggregates

The four-year field experiment exhibited a significant regression between enzyme activities and macroaggregates (>250 μm) (Table 3). Compost significantly increased the activities of C cycle enzymes in the 0–20 cm bulk soil (Fig 6b), with the exception of enzymes associated with aggregates (Fig 5). As the consequence of microbial growth and activity, soil C was strongly influenced by microorganisms during its formation and transformation. Microorganisms could continuously convert plant residue C into soil organic C in the form of metabolites (such as extracellular enzymes, extracellular polymers, etc.) or residues (cellular components from both living and senesced biomass) after repeated growth and metabolism. However, Ding et al. [45] indicated that the combination of soil organic matter and minerals also decreased the chance of microbial contact with plant residues during the agglomeration of soil particles, and the aggregates associated with organic C can only be utilized by microorganisms after the aggregate-crushing process. In the present study, the application of manure fertilizer appeared to synchronously promote the formation of macroaggregates and the activities of cellulase and urease, which are indicators of the catalytic strength of cellulase and urea, respectively, in macroaggregates. While organic fertilizer decreased the urease activities in the silt and clay fraction or microaggregates and invertase activities in macroaggregates (Fig 5). Redundancy analysis (RDA) also revealed that the FRM treatment only benefitted urease activities in macroaggregates (Fig 6d). Thus, with the formation of macroaggregates, the transformation of nitrogen and urea was also strengthened. This result could contribute to the practice of manure fertilizer according to the redundancy analysis results (Fig 6).

To date, concerning the roles of microorganisms in controlling terrestrial C fluxes, there are two critical contrasting points: promoting the release of C to the atmosphere through their catabolic activities and preventing release by stabilizing C into a form that is not easily decomposed [46,47]. Through a comparative analysis of the changes of soil aggregates and organic C composition caused by different fertilization treatments in the present research, the organic fertilizer treatment (FRM) significantly strengthened the structural stability of macroaggregates and significantly stabilized more stable organic C. Carbon cycle enzymes might be key controls on soil structure and function because of the intimate connection between enzyme activities and macroaggregates and between organic C composition and aggregates (Tables 2 and 3). Therefore, the addition of organic manure intensified the synergistic effect among aggregates, organic C and carbon cycle enzymes, and as a final result, more organic C was sequestered through a series of physical aggregation and the conversion of exogenous organic materials.

## Conclusions

The results obtained demonstrate increased accumulation of organic C in soil following organic fertilizer treatment. Compost amendment significantly increased the organic C content in soil by increasing organic C in macroaggregates (>250 μm), whereas the increase in organic C in soil to which chemical fertilizer was added was mainly because of the enhancement in the organic C content in microaggregates (<250 μm). The accumulation of organic C was mainly due to an increase in the iPOM subfraction within microaggregates and macroaggregates because both iPOM and organic C in the silt + clay subfraction were rather stable. The magnitude of the increased amounts of carbon cycle enzymes in aggregates or 0- to 20-cm bulk soil varied with fertilizer practices. The application of compost fertilizer significantly increased the cellulase and urease activities in macroaggregates but significantly decreased the invertase activities in macroaggregates. In particular, the process of mutual promotion between carbon cycle enzymes and macroaggregates might be strengthened by compost application, and the synergistic effect significantly increased soil organic C sequestration. Supplementation with organic manure such as compost is more beneficial to maintaining soil fertility production than a single application of chemical fertilizer in an intensively cultivated Vertisol.

## Supporting information

S1 Dataset (XLSX)
